# Cultural animation in health research: An innovative methodology for patient and public involvement and engagement

**DOI:** 10.1111/hex.12677

**Published:** 2018-03-12

**Authors:** Mihaela Kelemen, Emma Surman, Lisa Dikomitis

**Affiliations:** ^1^ Keele Management School/Community Animation and Social Innovation Centre Keele University Staffordshire UK; ^2^ School of Medicine/Research Institute for Primary Care and Health Sciences Keele University Staffordshire UK

**Keywords:** arts‐based methodologies, collaborative research, cultural animation, empowerment, health research, participatory research, patient and public involvement and engagement

## Abstract

**Background:**

A significant challenge in Patient and Public Involvement and Engagement (PPIE) in health research is to include a wide range of opinions and experiences, including from those who repeatedly find themselves at the margins of society.

**Objective:**

To contribute to the debate around PPIE by introducing a bottom‐up methodology: cultural animation (CA). Cultural Animation is an arts‐based methodology of knowledge co‐production and community engagement which employs a variety of creative and participatory exercises to help build trusting relationships between diverse participants (expert and non‐experts) and democratize the process of research.

**Design:**

Three CA full‐day workshops for the research project “A Picture of Health.”

**Participants:**

Each workshop was attended by 20‐25 participants including 4 academics, 5 retired health professionals who volunteered in the local community and 15 community members. Participants ranged in age from 25 to 75 years, and 80% of the participants were women over the age of 60.

**Results:**

The CA workshops unearthed a diversity of hidden assets, increased human connectivity, led to rethinking of and co‐creating new health indicators and enabled participants to think of community health in a positive way and to consider what can be developed.

**Discussion:**

Cultural animation encourages participants to imagine and create ideal pictures of health by experimenting with new ways of working together.

**Conclusion:**

We conclude by highlighting the main advantages to PPIE as follows: CA provides a route to co‐produce research agendas, empowers the public to engage actively with health professionals and make a positive contribution to their community.

## 1 INTRODUCTION

Patient and Public Involvement and Engagement (PPIE) is currently an integral part of both health‐care services and health‐care research in the UK.^1^, ^2^, ^3^, ^4^, ^5^ Recent research suggests that PPIE results in enhanced quality and appropriateness of research, in all stages of research programmes.^6^ Indeed, fostering robust PPIE is now at the heart of health‐related research and it is widely acknowledged that patients’ voices need to be heard and their concerns in the design and implementation of both health services and health research need to be taking more seriously. A significant challenge in PPIE is thus to include a wide range of “voices,” opinions and experiences, not only those from established groups whose members are practised in offering their opinions but also from those who repeatedly find themselves at the margins of society. A growing scholarly and public debate has emerged on how this is to be done most effectively rather than as a tick box exercise.

Approaches to PPIE often follow a standard process of inviting small groups of patients for a discussion session/focus group prior to embarking on the study and at regular intervals throughout the study. It remains a challenge to truly involve patients as team members “due to their location outside the health professions, and the social, spatial and temporal organization of their inclusion”.^7^ A cultural animation approach can meet such challenges in that it forges trusting, inclusive relationships between the researchers, health professionals, patients and the public.

Cultural animation is a participatory arts‐based and embodied methodology of community engagement and knowledge co‐production that draws on the everyday experiences of ordinary people and their creative abilities to achieve individual and collective goals.^8^, ^9^ CA was developed and pioneered in the UK by New Vic Borderlines (the outreach department of the New Vic Theatre, Newcastle‐under‐Lyme, UK) in collaboration with the Community Animation & Social Innovation Centre (CASIC) at Keele University.^10^

The methodology helps to build trusting relationships between participants by inviting them to work together in a series of workshop activities which may be new to them but which draw on their life‐experiences. Rather than relying solely on the written word, ideas are also explored through actions and images. Outputs usually include creative products such as songs, poems, short plays, puppets^11^ in addition to a more conventional data set. Consequently, a wider and more diverse audience can engage with the findings of the research.

A typical CA workshop includes a mixture of creative tasks, embodied activities and small group discussions to explore key themes (i.e the topic of the research). The workshop will begin with a series of name games, designed to put people at ease with each other, building on the idea that when people are not static, more participatory ways of approaching and solving problems are possible. These activities along with the creation of various artefacts, to which everyone's contribution is equally important, helps to dissolve hierarchies and traditional barriers associated with professional expertise. By “giving life to” the dynamics of everyday life through these activities, cultural animation encourages participants to reflect on the potential for change within themselves and their own communities. For a more detailed discussion of the theoretical underpinnings and the strengths and limitations of CA, see a recent study on community responses to the 2011 tsunami in Japan.^12^

While the benefits of participatory art programmes and initiatives on health and well‐being are well documented in the literature,^13^, ^14^ participatory arts‐based methodologies for researching with public and patients are yet to become mainstream. This article extends the extant discussion by articulating for the first time the opportunities that cultural animation methodologies can bring to PPIE activities in health‐focussed research. The methodology has been extensively used by the authors to co‐produce knowledge with communities in the UK and abroad (in Japan, Greece, Canada, France, the Philippines) on topics such as food poverty, communities in crisis, marketplace exclusion, disability, volunteering, social innovation, community leadership and health in the community.^15^ Here, we discuss the potential and challenges of using CA in health and well‐being research, by illustrating how we used this methodology to co‐create a health agenda with local communities in Stoke‐on‐Trent (UK).

According to Public Health England, the health of people in Stoke‐on‐Trent is generally worse than the average for England. Stoke‐on‐Trent is one of the 20% most deprived local authorities in England, and about 26% (13 300) of children live in low‐income families.^16^ Life expectancy for both men and women is lower than the average for England, and health inequalities have widened in recent years. With residents described by official documents as “leading unhealthy lifestyles”,^17^ this project aimed to explore the responses of community members to claims such as these to identify what “good health” meant for them and finally what participants might do in response to this situation at a community and individual level. Our research project, A Picture of Health, was funded by the Connected Communities Programme, under a scheme on Knowledge Co‐creation and Co‐design that sought to “support innovation in the process of co‐creation and co‐design of research with communities and added value through cross‐disciplinary approaches to co‐creation and co‐design incorporating distinctive arts and humanities perspectives”.^18^

## 2 CULTURAL ANIMATION AS A PPIE METHODOLOGY IN HEALTH RESEARCH

Cultural animation is located within the broader field of creative methods^19^ and underpinned by an ethos of dissolving hierarchies within mixed‐background groups to enable and facilitate the co‐creation of knowledge and embodied learning. This is particularly pertinent in the context of health‐care research, as it brings together patients, policymakers, health professionals and health‐care managers. Through the use of drama exercises, art making, poetry and music, CA aims to create a space in which existing hierarchies are less dominant and boundaries are crossed. By regarding practical skills, experience and expert knowledge as equal, CA exercises embrace the view that knowing and doing are deeply interconnected and equally important in the co‐creation of health knowledge and the co‐design of health‐related solutions. The use of boundary objects,^19^, ^20^ such a post‐it notes, ribbons, plates, picture frames, cardboard, buttons, fabric, is an integral part of this methodology, for these objects help participants express individual and collective ideas and articulate them through different ways of knowing.^21^ The use of boundary objects increases human connectivity and builds up trust between members of the public, academics and health professionals.^22^, ^23^ They help redistribute the power between participants, providing both the opportunity and the authority for those involved to exchange different types of knowledge and co‐produce shared knowledge as we will see in the case study below (Box 1).

Box 1 **Pillars of cultural animation^27^**

1

1. Draws on the everyday experiences of people and their creative abilities to make sense of the world.
2. Builds up trusting relationships between participants by inviting them to work together in activities which may be new to them but which rely on their life‐experiences.
3. When people move about and complete tasks together, it facilitates new ways of seeing and thinking.
4. Boundary objects (everyday objects) are central to the collaboration and communication between academics, medical practitioners and members of the public.
5. Common sense, academic expertise and practical skills are valued in equal measure.
6. Knowledge and experiences are articulated in actions, images, installations as well as via the written word.
7. The cultural animateur acts as a facilitator.
8. Pioneered in the UK by New Vic Borderlines and the Community Animation and Social Innovation Centre at Keele University.

The research project “A Picture of Health” aimed to co‐design both the research questions and the research findings in line with the ethos of the connected communities research scheme under which it was funded. The research process included 3 CA workshops held at the New Vic Theatre. The workshops started at 10 am and ended at 4 pm, and lunch and drinks were provided. Recruitment was facilitated through the relationships built up by New Vic Borderlines with statutory organizations and local communities through its outreach work. Participants were invited to attend if they were interested in the health and well‐being of local communities and were not compensated for their time and participation. They were not required to sign up to all 3 workshops. Each workshop was attended by 20‐25 individuals and included 4 academics, 5 retired health professionals who volunteered in the local community and 15 community members most of whom were retired. Participants ranged in age from 25 to 75 years, and 80% of the participants were women over the age of 60 who were retired and volunteered at the New Vic Theatre. The workshops were facilitated by the 3 theatre practitioners^21^ (hereafter referred to as CA facilitators). Workshop 1 was used to co‐design the issues to be further unpacked in the course of the research project, namely “What is a healthy community?” explored in the first workshop, “Ageing well and dying at home,” explored in the second workshop and “How communities and government could work together to improve health” explored in the final workshop. The workshops were digitally recorded and transcribed, which resulted in over 50 pages of data. The authors also took photographs during the workshops and carried out follow‐up qualitative interviews (see Box 2) with 15 participants (3 men, 12 women) to assess the impact the CA workshops had on themselves and their immediate communities. The interviewees consisted of one retired nurse, one retired GP and 3 retired NHS senior managers along with 10 community members who, in addition to being users of health services in their own right, volunteer and work with asylum seekers and people with disabilities in their local communities. We employed narrative analysis^24^, ^25^ to analyse the interviews, the field notes and the visual data. The emphasis was on the co‐construction of meaning between the researchers and participants. While being involved in the making of artefacts, contributing and listening to the conversations, we compared what was being said and to our own personal understandings aiming to piece together and make sense of each story in its own context. The process of “data gathering” and “analysis” was therefore concurrent rather than separate, and as such, the story of the researcher was one of the many that have been constructed and orchestrated in the analysis. The ethical approval for the research project was granted by Brunel University on 13 February 2013. Workshop participants were fully briefed prior to participating via a Participant Information Sheet as well as further verbal explanations by the research team members. Informed, written consent was obtained via a consent form.

Box 2 **Interview topic guide**

1

1. What was your definition of health before and after the workshops?
2. If that changed, what has prompted the change?
3. How should health be measured and by whom?
4. Do you think your community is willing/able to make changes to its health in near future?
5. What is needed to ensure that communities have more of a say in what counts as good health?
6. What are the benefits and challenges of cultural animation? Please provide examples of both.
7. Is this an approach you would use in your current volunteering work? Why?
8. How do you feel about the future of health for people in Stoke‐on‐Trent?

## 3 RESULTS

### 3.1 Workshop 1: what is a healthy community?

**Figure 1:**
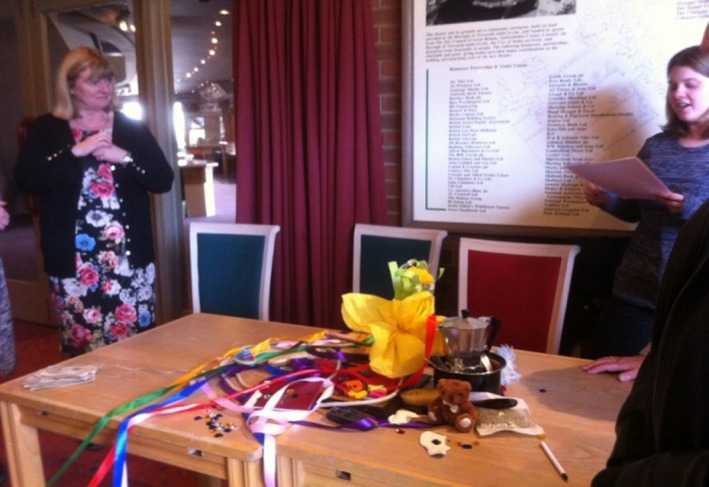
Art installation representing a community that has great health

The first workshop aimed to co‐design the frames of reference for the research by exploring the meanings of a healthy community. The starting point was a presentation of the current health statistics for Stoke‐on‐Trent given by a retired NHS chief executive. His presentation sparked a debate about how (ill) health is defined and measured by government. Participants took issue with the picture painted by these statistics and did not recognize their communities in these official reports. Participants were then randomly split into 2 groups and asked by the CA facilitator to work together to create an art installation of “a healthy community.” As part of this task, they were encouraged to use the objects brought to the workshop by the CA facilitators to convey what health feels, looks, sounds and tastes like in their immediate communities. One group worked with colourful ribbons, buttons and empty frames to create the installation are presented in Figure 1.

This “healthy community” was perceived as a dynamic, open‐minded, happy community: “this is a messy community which is close knit, having lots of fun together, which is always changing and is open to new members and ideas” (field diary author 1). It was described by a retired NHS manager as a “jewel of a community, with good neighbours, with a thriving industry, vibrant and colourful” (field notes). A member of the local public and NHS‐user added: “It is an emergent community: we kept changing it, because the community changes and we wanted to capture this change.”

**Figure 2:**
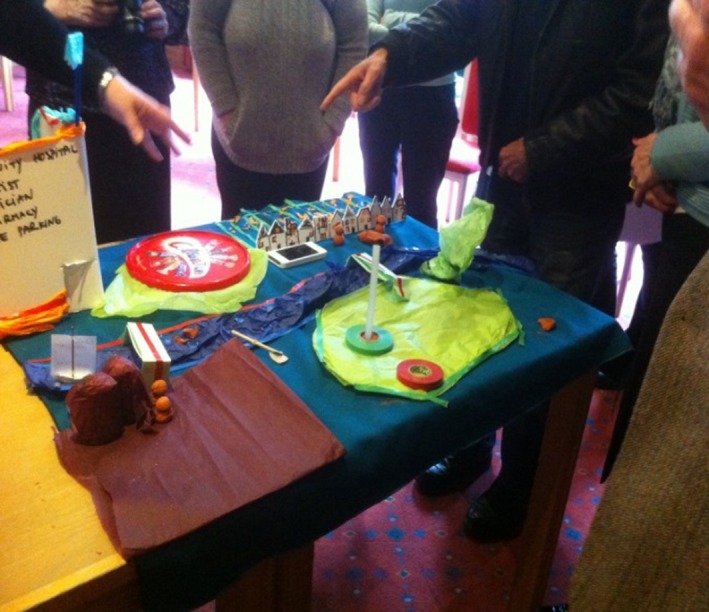
Art Installation of a community which has great health

The second group created an orderly community out of cardboard, empty packs and straws (Figure 2).

This “healthy community” was a neat locality with terraced houses, allotments, bike paths, a community centre and free parking. The spirit of this community was described by the group in poetic form:

You see here the river, the open green spaces, other people who speak to you

You hear children playing, people talking, the sound of water, the noise of industry

You feel good and safe

People grow their food, they go to work, play, cycle, they are active

You smell cut grass, herbs and flowers from the allotments, water, food being cooked.

The objects used to create the art installations acted as boundary objects which helped participants make sense of what counted as health to them and share these ideas with other group members. By working together in this embodied and creative way, it was possible for individuals from different backgrounds to connect, communicate and achieve some consensus around a topic that generated strong feelings and emotions. In subsequent interviews, participants reflected on their collective work, commenting that these “healthy communities” were not fantasies in that aspects of such communities already existed or could easily exist if people simply changed their perception and understanding of health. In contrast to the perception of Stoke‐on‐Trent as deeply unhealthy that was given via the official statistics, the CA activities identified and highlighted positive aspects of the local communities represented in the workshop. One community member said in the follow‐up interview: “We don't need to associate the GP [General Practitioner] with health. The GP wants to deal with ill health issues that can be cured through medication and operations.” This was reiterated by another member of the public: “Communities should come up with their own measures of health. What is measured in health is far from day to day life and the solutions are so huge that communities do not think they could have an influence” (retired NHS manager in interview). The workshop concluded with participants agreeing the themes for the subsequent 2 workshops and proposing their own measures for good health in terms of creativity and connectivity in the community. This included the creation, identification and quantification of participation in activities such as choirs, drama groups, sports clubs and tending allotments.

### 3.2 Workshop 2: ageing well and dying at home

The second workshop comprised of 20 participants, with 5 who did not attend the first workshop. The CA facilitator invited participants to create an ideal community for older people—a theme established in workshop 1—by identifying how such a community would make a difference to their lived experience but also to dying in one's own home.

The workshop started with participants listening to a recorded story about an older woman played out in a reconstructed room. Participants described the feelings evoked by the story in the following terms: the old woman lives on her own, it is cold in her house; she is a widow, she is frightened and powerless; there is regret; she is lonely; she mentions the weather as an impediment for getting about; her age is a mental construction; she feels isolated; she is reminiscing about old days; her life feels pre‐destined; she is frustrated she did not help her friends; there is anxiety on her mind that she will die in hospital and not home (from field notes).

A retired NHS doctor said that in the UK people tend to die in hospitals, yet many people want to die at home and they are not able to. A community member lamented that there are poor end‐of‐life provisions in place and felt people had no choice in the matter.

**Figure 3:**
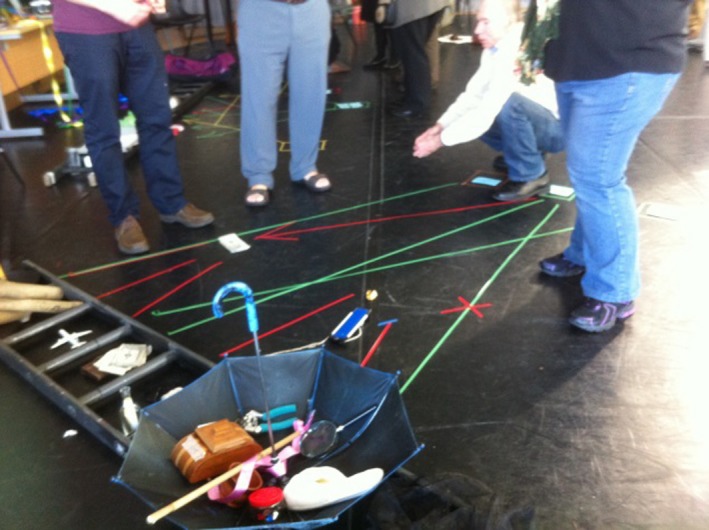
Dying at home installation

After this discussion, participants were asked by the CA facilitator to imagine a community that would make a real difference to the lived experience of older people living and dying in their communities. The ensuing installation about communities that could support people dying at home can be seen in Figure 3.

The arrows point to the human and cultural assets that already exist within communities and would make it possible to die at home with dignity. One retired NHS manager suggested Maslow's hierarchy of needs as a starting point and explained to the others its elements: physiological needs, safety, belonging, esteem and self‐actualization. The participants then selected objects to signify the importance of each of these levels. In addition to the various objects chosen to convey the physiological needs, they also selected objects which spoke of the importance of belonging and self‐esteem. These included a message in a bottle and an umbrella to highlight the importance of communication and support and a candle to signify dignity. Unlike the depiction by Maslow, the participants felt these needs should not be seen as hierarchical. The group concluded that all these needs could be met by the community, but that it would require a shift in how people (including themselves) behave towards one another in a highly individualistic world. At the end of the workshop, one CA facilitator commented: “Nobody focused on external services, people spoke about what they themselves can bring to this process. You've all described journeys that are perfectly attainable and do not require external resources.” (Field notes).

### 3.3 Workshop 3: the relationship between the communities and the government

In the final workshop, participants were asked to draw on the activities and reflections from the first 2 workshops to communicate their own “picture of health” to the rest of the world, including government. This was achieved by completing 2 activities: (i) using social media as a framework to convey their findings to a general audience and (ii) producing a presentation to government in the form of a human tableaux.

**Figure 4:**
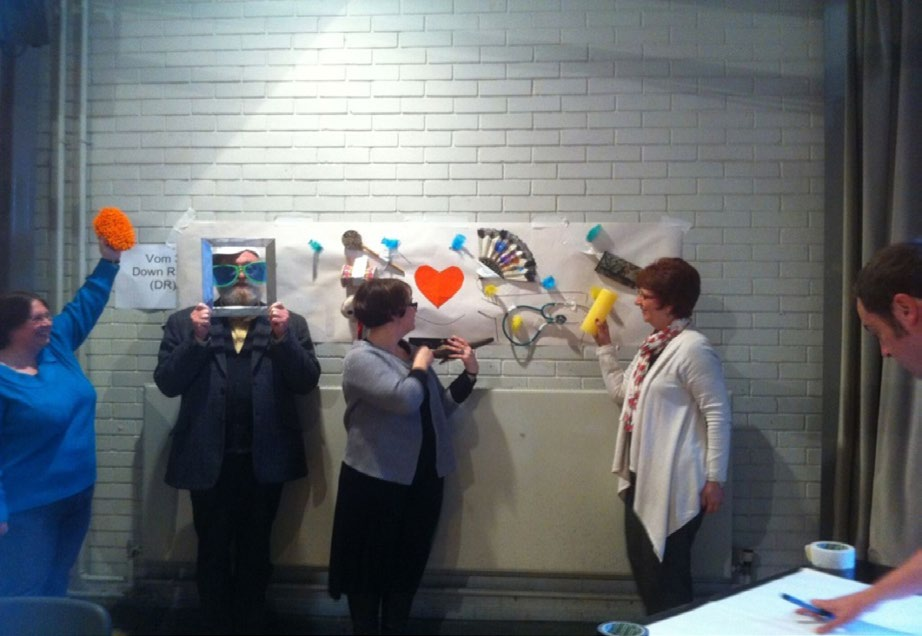
Facebook page art installation

For the social media, exercise participants were asked to construct a 3‐dimensional Facebook page. One groups’ creation can be seen in Figure 4, in which the heart signified “friendship,” the candle “hope,” the stethoscope “access to the GP” and the sunglasses “leisure and rest time” as elements of health in the community.

The selection of objects to create the installation allowed people to find and express a creative self which led to increased levels of energy in the room and to productive discussions and effective problem‐solving. After the 3‐dimensional Facebook page was constructed, the group was asked to produce their first “post.” To encourage the group to distil their thoughts into concise wording, they were asked to create their post in the form of a Haiku. These are 2 “posts” written by the groups:

A Sad man called Joe

Opened his creative mind

To help those in need

Try to make a difference

Bring some sunshine

Come on folks

The second part of the exercise involved the groups composing a Tweet about good health. This was done in the form of a cinquain (poems comprising 5 lines): the first verse has one word (the title), the second verse has 2 words describing the title, the third verse has 3 actions that make the title happen, the fourth verse has 4 words describing feelings associated with the title, and the final verse has one word which is an alternative word for the title. One group composed this poem about community creativity:

Creativity

Beautiful, Inspirational

Engage, Think, Enjoy

Calm, Love, serene, happy

Wholeness

Another group started from the pronoun “We” and ended with a message of togetherness.

We

United, caring

Aspire, achieve, enjoy

Calm, proud, content, valued

Together

A participant described his community as follows: “We are a happy community. We like to have fun, the sun is shining on our community. The candle helps us to go in the right direction, it is a community for different ages, we offer money advice to people, our dreams can come true. The GP practice has a participatory group. We organise events such as youth clubs, coffee mornings, mother and toddler, learn how to cook” (field notes).

**Figure 5:**
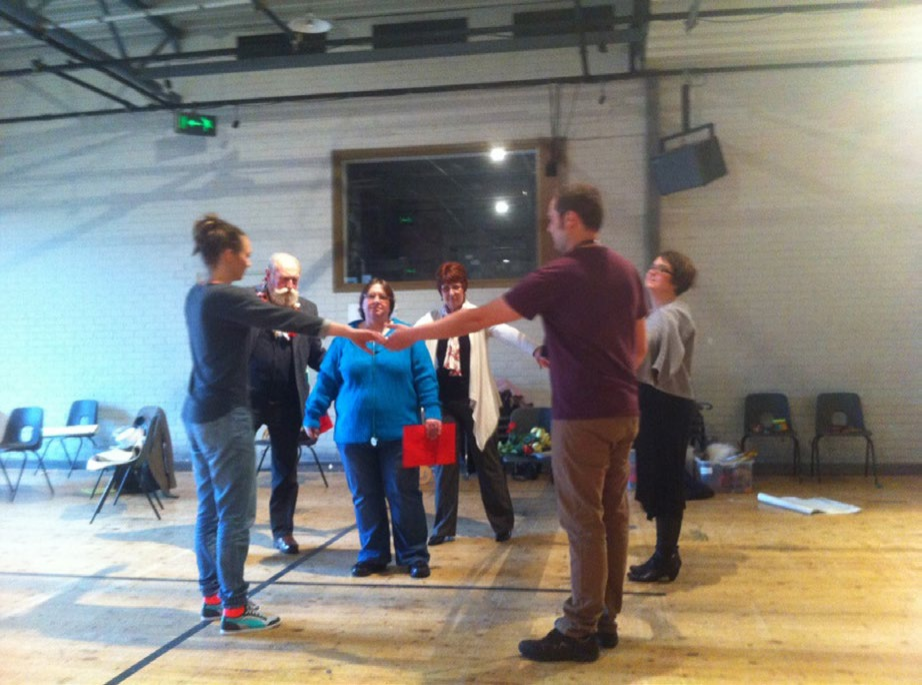
Presentation to the government

In the final part of the workshop, each group was invited to enact these poems as if presenting their findings to a government (played by other participants). One group chose to do this by holding hands and reciting the poems together (see Figure 5).

The aim of the presentation was to convince the fictional government that what is going on in the community is worth supporting. The narrative of one of the groups went as follows:

One of four people will suffer mental illness at some point in their life but you don't know who is going to be. It could be anyone, it could be you. We think we have the answer in our community.

(field notes)

They then sang a song in a very upbeat rhythm: “There was a sad man called Joe, who did not know where to go. Who are we? Community, creativity, diversity, equality, inclusivity in Stoke on Trent, all in our backyard” (field notes).

One of the participants approached the table where those playing the part of the government were sitting and said: “Here is an advance payment of what we saved you so far (fake money is handed over). But doing this with your support will save you half a million per year. And that's just a start. If it works for mental health, it will work for all health.” The participants playing the government role then agreed to support the proposed community‐based health initiatives.

Despite the negative indicators of health for Stoke‐on‐Trent, at the end of the research project, participants agreed that their own communities could make a difference to their own health. New health indicators were co‐created such as creativity, connectivity, inclusivity and acceptance of difference. Participants felt that such indicators are more relevant than currently used indicators which actually measure ill health according to parameters such as teenage pregnancy, obesity levels and alcoholism.

When the participants were presented with the official statistics on health at the beginning of the first workshop, although they did not feel that the numbers reflected their own experience of their communities, they were not able to articulate an alternative to challenge this. By the end of the final workshop, it appeared that participants had acquired the resources to do this. One community member said: “I want to start a revolution. The government is funding the ill health service.” (field notes). A retired medical professional re‐enforced this point of view by asking: “Why can't good health be about the number of drama clubs, choirs, how many people go bowling or go to a club together?” It became clear that for the participants, healthy communities are those that support the dreams and aspirations of their members and that health is about human relationships rather than about doctors, hospitals and financial resources.

### 3.4 Feedback on cultural animation

At the end of the final workshop, the CA facilitator asked participants for feedback on the process and the techniques of cultural animation. Participants said that CA “sets people to open up as they are not overwhelmed by words.” The methodology also “visualises change, you feel like you've already started the process of change.” The process was also described as “truly democratic,” “empowering,” “honest,” “practical” and “valuable” (field notes). The reflexive diary of the author states: “I feel I have learnt so much about my health and my community, but more importantly I've learnt about myself, how I am perceived and how I can relate to people from all walks of life. I don't think I could ever go back to the way I used to do research” (personal diary, March 19, 2014).

The participatory methodology used in this project unearthed a diversity of hidden assets: skills and experiences that individuals possessed already but either they may not have been aware of, or not had the confidence to share them; exchanges that would lead to new opportunities; empty buildings that could be used for communal purposes; relationships that could be shared with others; networks, organizations and clubs that could support people in need; access to IT in public spaces and so on. One of the retired nurses who currently volunteers in the community said in a follow‐up interview: “Refugees and asylum seekers have so much to offer but we don't let them. I can point people in the right direction, teach them how to get into the system and use their skills and knowledge, in return they teach me how to cook and one of them even babysat my grandson.”

Cultural animation also led to increased human connectivity between diverse parties, as these 2 interview quotes from community members illustrate:

This methodology helped me to open up and to become more empathetic to other people's problems and points of view. I am sometimes overwhelmed by words but the fact that we could speak through objects was very helpful to reach out to the others.

People were just in, with all of their being. And we had little to nothing of the usual stuff to work on …what people then say about themselves, their motives and purposes is all caught up in the creation of a task and practical responses in the moment. There was no status and no history just the present and an imaginary future. We were in it together.

## 4 DISCUSSION

The CA techniques used in the workshops encouraged all participants to imagine and create “healthy communities” (of the most immediate and easily available resources) by experimenting with new ways of working together. These ways of working promoted a form of democratic communication that went beyond words and relied on mutual trust and reciprocal help. It is precisely this aspect that could benefit PPIE processes in challenging prevailing social norms such as the view that “doctors know best what good health is” (field notes, workshop 1). The process of interaction allowed new and more creative dialogues to unfold. Of these dialogues, different types of relationships emerged which ensured that the experts and non‐experts shared the power, the resources and the ideas equally in the conversation of research. This is crucial for PPIE processes if they are to truly give voice to patients and the public and establish an environment of equality.

Our findings from this research project suggest that cultural animation can:

1. unearth hidden assets in the community,
2. increase human connectivity,
3. rethink the meaning of health and co‐create new health indicators grounded in day‐to‐day experience, and
4. enable people to think of their communities in a positive rather than negative way and to consider what is already in place and what can be developed rather than the ways in which statistically they are seen to have failed.

To conclude, the cultural animation methodology has the potential to be used in PPIE activities. This project, the first to utilize CA as a form of PPIE in health research, has identified 3 main methodological advantages as follows:

Firstly, the CA methodology facilitates genuine engagement with the research topic that goes beyond asking about and responding to concerns by stimulating contributions from all participants on the basis of reciprocity, respect and openness to the process. Thus, PPIE participants could be, indeed should be, involved in the research process through observing, discussing and enjoying the activities taking place around them. They can contribute as much or as little as they feel able to for it is made clear to them that are no correct or incorrect ways of approaching an issue.

Secondly, CA allows for different types of connections to form. These human connections are held together by boundary objects which are infused with personal and collective meanings and trigger the exchange of knowledge, experiences, feelings and emotions. The request to produce performances and create installations within short periods of time ensures that participants respond to any requests made by the others effectively and generously. This leads to a sense of camaraderie and to a change in one's perceptions about others and the topic discussed. When translated to a PPIE environment, CA can develop a sense of collective consciousness and empowerment to take action at an individual level and collective level.

Thirdly, by employing CA, deep beliefs about what is possible are revealed. The focus is not on what is lost in the community but on the future, on what can be changed. When applied to PPIE processes, CA can enable patients to articulate personal and communal ambitions with regard to health and create a common agenda for change in a collaborative bottom‐up fashion.

In addition, CA can ensure that the reporting of the research findings which remains problematic in PPIE research^5^ can take place both via conventional scholarly outlets, such as conference presentations, journal papers and academic books, and in venues where the wider public can more easily engage with the findings (public exhibitions, performances, podcasts and blogs).

It is thus clear that cultural animation goes beyond the ethos and promises of the existing PPIE initiatives by facilitating the development of knowledge co‐production practices which are central to the empowerment of the public and by encouraging collaborative relationships with health experts which are based on equality and mutual respect, thus representing a more genuine form of public involvement. Testimony to the strengths and potential of this methodology is the recent award made by AHRC and MRC for a global health project—SOLACE—in which CA is the central PPIE methodology.^26^ As this is the first study of its kind, it is crucial that more studies are conducted on the workings and impact of CA on PPIE processes to distil best practices and develop conceptual insights that could refine and extend existing theoretical frameworks.

## CONFLICT OF INTEREST

The authors have no conflict of interest to declare.
